# Distributions of Globotriaosylceramide Isoforms, and Globotriaosylsphingosine and Its Analogues in an α-Galactosidase A Knockout Mouse, a Model of Fabry Disease

**DOI:** 10.1371/journal.pone.0144958

**Published:** 2015-12-14

**Authors:** Hideaki Sueoka, Mikio Aoki, Takahiro Tsukimura, Tadayasu Togawa, Hitoshi Sakuraba

**Affiliations:** 1 Genomic Science Laboratories, Sumitomo Dainippon Pharma Co., Ltd., Konohana-ku, Osaka, Japan; 2 Department of Functional Bioanalysis, Meiji Pharmaceutical University, Kiyose, Tokyo, Japan; 3 Department of Clinical Genetics, Meiji Pharmaceutical University, Kiyose, Tokyo, Japan; Weizmann Institute of Science, ISRAEL

## Abstract

Fabry disease is caused by deficient activity of α-galactosidase A (GLA) and characterized by systemic accumulation of glycosphingolipids, substrates of the enzyme. To gain insight into the pathogenesis of Fabry disease based on accumulated substrates, we examined the tissue and plasma distributions of globotriaosylceramide (Gb3) isoforms, and globotriaosylsphingosine (lyso-Gb3) and its analogues in a *GLA* knockout mouse, a model of Fabry disease, by means of liquid chromatography-mass spectrometry and nano-liquid chromatography-tandem mass spectrometry, respectively. The results revealed that the contents of these substrates in the liver, kidneys, heart, and plasma of *GLA* knockout mice were apparently higher than in those of wild-type ones, and organ specificity in the accumulation of Gb3 isoforms was found. Especially in the kidneys, accumulation of a large amount of Gb3 isoforms including hydroxylated residues was found. In the *GLA* knockout mice, the proportion of hydrophobic Gb3 isoforms was apparently higher than that in the wild-type mice. On the other hand, hydrophilic residues were abundant in plasma. Unlike that of Gb3, the concentration of lyso-Gb3 was high in the liver, and the lyso-Gb3/Gb3 ratio in plasma was significantly higher than those in the organs. The concentration of lyso-Gb3 was apparently higher than those of its analogues in the organs and plasma from both the *GLA* knockout and wild-type mice. This information will be useful for elucidating the basis of Fabry disease.

## Introduction

Fabry disease (OMIM 301500) is characterized by storage of glycosphingolipids in organs and tissues throughout the body, resulting from deficient activity of α-galactosidase A (GLA, EC 3.2.1.22) [1,2]. This lysosomal hydrolase encoded by the *GLA* gene (locus: Xq22.1) catalyzes the removal of terminal α-linked galactosyl residues from glycosphingolipids, predominantly globotriaosylceramide (Gb3). Patients with Fabry disease show progressive accumulation of Gb3 and related glycosphingolipids in the peripheral nerves, skin, eyes, intestine, kidneys, and heart and vascular systems, leading to systemic disorders, although they exhibit heterogeneous manifestations due to harbored gene mutations and gender [1,2]. Gb3 consists of a sugar chain (galactoseα1-4galactoseβ1-4glucose) linked with a ceramide moiety that is composed of sphingosine and various fatty acids. Therefore, it is predicted that there are many Gb3 isoforms in organs and tissues due to the respective metabolic pathways.

**Fig 1:**
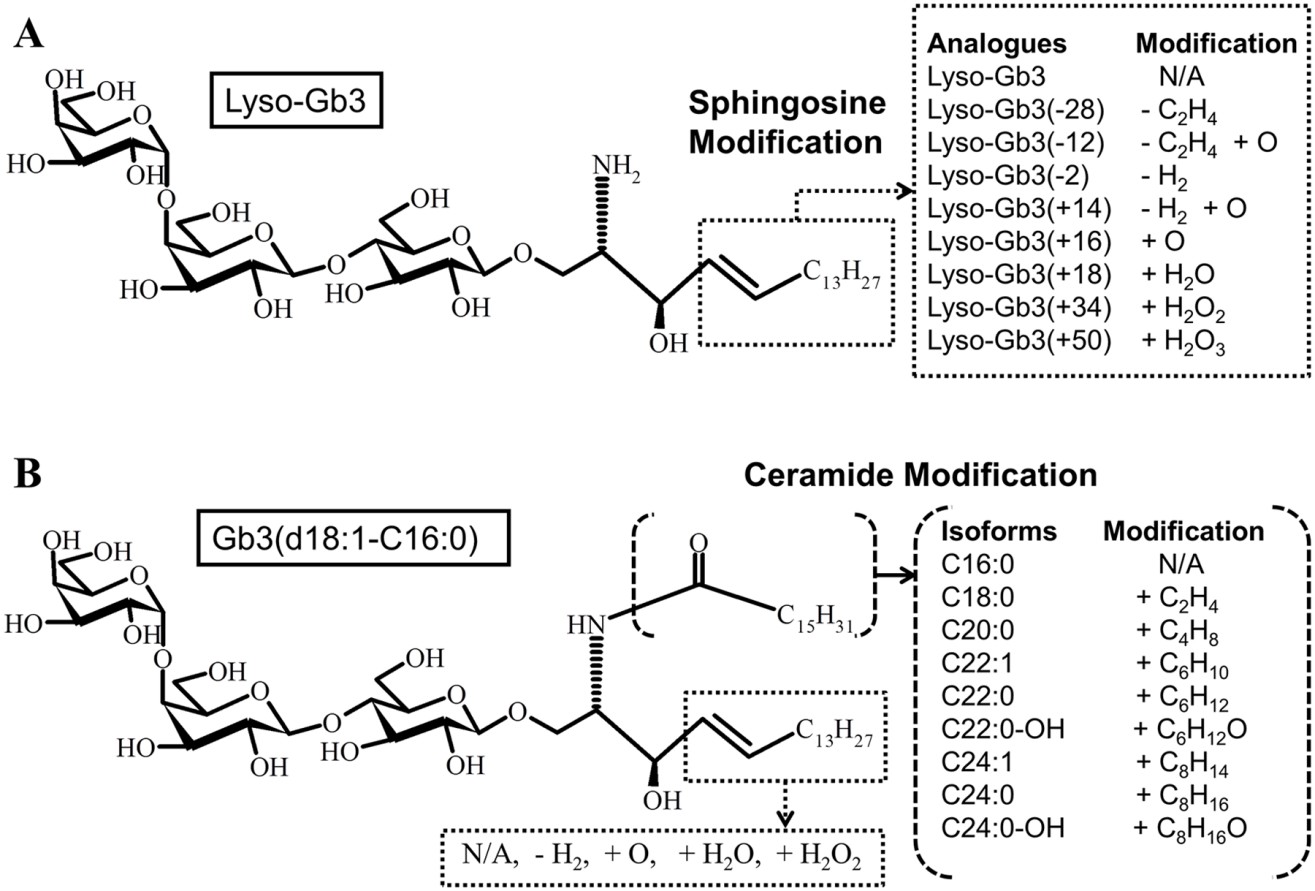
Lyso-Gb3 analogues and Gb3 Isoforms. (A) Chemical structure of Lyso-Gb3 and sphingosine modification of lyso-Gb3 analogues. (B) Chemical structure of Gb3(d18:1-C16:0) and ceramide modification of Gb3 isoforms having various fatty acids linked with various sphingosine moieties corresponding to lyso-Gb3 and four analogues.

Recent studies revealed that the deacylated form of Gb3, globotriaosylsphingosine (lyso-Gb3), also accumulated in plasma and urine of Fabry patients, and lyso-Gb3 is expected to be a biomarker of this disease for diagnosis, monitoring of disease progression and assessment of therapeutic efficacy [3–6]. Lyso-Gb3 analogues having various sphingosine modifications are also reported to be biomarkers of the disease [7,8] (Fig 1A).

Considering the above, it is thought that such glycosphingolipid accumulation is deeply associated with the pathogenesis of Fabry disease. Although Gb3 itself is a cell component, its excessive accumulation causes endothelial dysfunction [9–11] and nephropathy through increased expression of cytokines [12]. Increased lyso-Gb3 also leads to injury to glomerular podocytes [13] and sensory neurons [14], and also causes proliferation of smooth muscle cells, which may be related to the vascular defect in Fabry disease [3]. However, there remained many problems to be solved for understanding the pathogenesis of Fabry disease, i.e., differences in species of glycosphingolipids accumulated in organs, and their influence in each organ and tissue.

Recent development of sensitive assay methods enabled us to detect Gb3 isoforms due to heterogeneous fatty acids with various sphingosine moieties (Fig 1B), and to measure small amounts of lyso-Gb3 and its analogues by means of liquid chromatography-mass spectrometry (LC-MS) [15] and nano-liquid chromatography-tandem mass spectrometry (nano-LC-MS/MS) [16], respectively.

So far, at least two strains of *GLA* knockout mice have been established [17,18], and which are widely used as animal models of Fabry disease [19–25]. In this study, we examined the distributions of Gb3 isoforms, and lyso-Gb3 and its analogues in GLA knockout mice using LC-MS and nano-LC-MS/MS to obtain information for an insight into the pathogenesis of Fabry disease.

## Materials and Methods

### Animals

The C57BL/6 *GLA* knockout mice [17,26] were denoted by A.B. Kulkarni and T. Oshima (National Institutes of Health, Bethesda, MD), and a colony was maintained at Meiji Pharmaceutical University. In this study, 8-month-old GLA^(-/0)^ males and GLA^(-/-)^ females were used. Given the genetic background of the Fabry mouse colony, 8-month-old C57BL/6 wild-type mice were used as controls. Mice were housed two to three per cage (size: 15cm×25cm×12.5cm) with woodchips bedding, and food and water were provided *ad libitum*. Blood samples were collected from the orbital venous plexus under anesthesia with diethyl ether. Then, the mice were sacrificed under pentobarbital-anesthesia, and their kidneys, liver, and heart were harvested for the biochemical analysis. The study involving mice was approved by the Animal Care and Use Committee of Meiji Pharmaceutical University.

### Reagents

Gb3 (mixture of various isoforms), Gb3(C16:0), Gb3(C17:0), and lyso-Gb3 were purchased from Matreya, LLC (Pleasant Gap, PA, USA). Stable-isotope labeled lyso-Gb3 (lyso-Gb3-IS), as an internal standard (IS), was synthesized according to a previous report [16] at Nard Institute, Ltd. (Kobe, Japan). Two stable-isotope labeled chemicals were used for lyso-Gb3-IS synthesis: L-Serine-1-^13^C (isotopic purity, 99%; Cambridge Isotope Labolatories, Inc., Tewksbury, MA, USA) and palmitoic acid-CD_3_ (isotopic purity, 99%; ISOTEC, Inc., Miamisburg, OH, USA). Therefore, lyso-Gb3-IS has one ^13^C and three deuteriums. For sample preparation and LC-MS/MS analysis, LC-MS grade isopropyl alcohol (IPA) and ammonium formate were purchased from Sigma-Aldrich Co., LLC (St. Louis, MO, USA). Phosphoric acid (H_3_PO_4_), LC-MS grade methanol (MeOH), and acetonitrile (ACN) were purchased from Kanto Chemical Co., Inc. (Tokyo, Japan), and chloroform and LC-MS grade formic acid (FA) from Wako Pure Chemical Industries, Ltd. (Osaka, Japan).

### Sample preparation

Gb3 isoforms were extracted from three organs (liver, kidneys and heart), and plasma of the wild-type (three males and three females) and *GLA* knockout mice (six males and six females). The organ tissues (20–100 mg) were homogenized in methanol using 3 mm Zr beads with a homogenizer (Micro Smash MS-100R; TOMY DIGITAL BIOLOGY CO., LTD., Tokyo, Japan). Each homogenate including 5 mg tissue was diluted with 600 μl of methanol containing 500 ng Gb3(C17:0), as an internal standard. Then, 300 μl of chloroform and 100 μl of water were added, and the crude lipids were extracted. In the case of extraction from plasma, 50 μl of plasma was diluted with 100 μl of water, and then 600 μl of methanol containing 500 ng Gb3(C17:0) and 300 μl of chloroform were added and mixed. Each mixture was centrifuged for 10 min at 14,000g, and then the supernatant was dried in an evaporator. Each residue was reconstituted in 200 μl of methanol and then used for conventional LC-MS/MS analysis. As standard samples, 100 μl of 0 to 100 μg/mL Gb3 (mixture of Gb3(C16:0) and Gb3 (various isoforms) = 1/1) in methanol was extracted following the same procedure.

Lyso-Gb3 and its analogues were also extracted from the organs and plasma of the wild-type (three males and three females) and *GLA* knockout mice (six males and six females). The homogenates including 5 mg tissue were diluted with 600 μl of methanol containing 1 ng lyso-Gb3-IS. Then, 300 μl of chloroform and 100 μl of water were added and mixed, and then the crude lipids were extracted. For extraction of the crude lipids in plasma, 20 μl aliquots of plasma samples were diluted with 100 μl of water. Then, 600 μl of methanol containing 1 ng lyso-Gb3-IS and 300 μl of chloroform were added and mixed. Each extract was centrifuged for 10 min at 14,000g, and then the supernatant was dried in an evaporator. Each residue was reconstituted with 1% H_3_PO_4_/MeOH, followed by transfer to an OASIS MCX cartridge (30mg, 60mm; Waters Corp., Milford, MA, USA). Extraction of lyso-Gb3 and its analogues by OASIS MCX was performed by the method described in our previous report [16]. As standard samples, 100 μl of 0 to 1500 ng/mL of lyso-Gb3 in methanol was extracted following the same procedure.

### Measurement of Gb3 isoforms by means of LC-MS/MS

For measurement of Gb3 isoforms, LC-MS/MS analysis was performed according to a previous report [27]. For conventional-LC, an Ultimate 3000 (Thermo Fisher Scientific, Inc., Waltham, MA, USA) was used. Hypersil Gold (150 mm x 4.6mm, 3um particles; Thermo Fisher Scientific) was used for reversed phase based chromatographic separation. Five micro liters aliquots of samples were injected. The column oven was set at 40°C. Solvent A consisted of ACN/MeOH/water (19/19/2), 20 mM ammonium formate and 5 mM formic acid, and Solvent B of IPA/water (100/1), 20 mM ammonium formate and 5 mM formic acid. A mobile-phase gradient was produced during a 35 min run: 0 min, 10% B; 25 min, 25% B; 25.1 min, 95% B; 30 min, 95% B; 30.1 min, 10% B; and 35 min, 10% B. The flow rate was 0.5 mL/min. A Q-Exactive mass spectrometer (Thermo Fisher Scientific) was used for the detection of Gb3 isoforms. Instrument calibration was performed before each analysis. The samples were injected into the mass spectrometer from 2 to 25 min by a switching valve. The targeted MS/MS analysis (HRPS) mode was selected for quantification of Gb3 isoforms. The ion spray voltage was set at 2500V in the positive ion mode. The first quadrupole was operated at 1.0 FWHM and the Orbitrap spectrometer at 70,000 FWHM. The AGC target value was set at 2E5, with a maximum injection time of 100 ms. The collision energy value was 40% for all the compounds of interest. The theoretical masses of Gb3 isoforms, which had various fatty acids linked with various sphingosine moieties corresponding to lyso-Gb3 and four analogues (Fig 1B), were targeted. The target masses and acquisition times are shown in Table 1. The precursor ions of Gb3 isoforms were sodium adducts. Fragment ions due to neutral loss of a single galactosyl fragment (162.05 Da) from the precursor ion were selected for quantification of Gb3 isoforms with our instrument (Table 1). The calculation for measurement of Gb3 isoforms was performed using the Quan Browser software (Thermo Fisher Scientific). The total Gb3 concentration was calculated from the sum of all the Gb3 isoforms we had detected.

**Table 1:** The target masses and acquisition times of Gb3 Isoforms.

No.	Formula	Sphingosine modification	Fatty acid moiety	Targeted Mass [m/z]	Start [min]	End [min]	Quantification Mass [m/z]
1	C_52_H_95_NO_18_	- H_2_	C16:0	1044.64	7	10.3	882.59
2	C_52_H_99_NO_20_	+ H_2_O_2_	C16:0	1080.66	7	10.7	918.61
3	C_52_H_99_NO_19_	+ H_2_O	C16:0	1064.67	7	10.9	902.62
4	C_52_H_97_NO_19_	+ O	C16:0	1062.65	7	11.6	900.6
5	C_52_H_97_NO_18_	N/A	C16:0	1046.66	7	12	884.61
6	C_54_H_99_NO_18_	- H_2_	C18:0	1072.67	7	12	910.62
7	C_54_H_103_NO_20_	+ H_2_O_2_	C18:0	1108.7	7	12.4	946.65
8	C_54_H_103_NO_19_	+ H_2_O	C18:0	1092.7	7	12.7	930.65
9	C_54_H_101_NO_19_	+ O	C18:0	1090.69	7	13.6	928.64
10	C_54_H_101_NO_18_	N/A	C18:0	1074.69	10.3	13.8	912.64
11	C_56_H_103_NO_18_	- H_2_	C20:0	1100.71	10.7	14	938.66
12	C_58_H_105_NO_18_	- H_2_	C22:1	1126.72	10.9	14.2	964.67
13	C_56_H_107_NO_20_	+ H_2_O_2_	C20:0	1136.73	11.6	14.6	974.68
14	C_58_H_109_NO_20_	+ H_2_O_2_	C22:1	1162.74	12	14.6	1000.69
15	C_56_H_107_NO_19_	+ H_2_O	C20:0	1120.73	12	15	958.68
16	C_58_H_109_NO_19_	+ H_2_O	C22:1	1146.75	12.4	15	984.7
17	C_56_H_105_NO_19_	+ O	C20:0	1118.72	12.7	15.8	956.67
18	C_58_H_107_NO_19_	- H_2_	C22:0-OH	1144.73	13	16.3	982.68
19	C_56_H_105_NO_18_	N/A	C20:0	1102.72	13.6	16.5	940.67
20	C_58_H_107_NO_18_	N/A	C22:1	1128.74	13.8	16.8	966.69
21	C_60_H_109_NO_18_	- H_2_	C24:1	1154.75	14	16.9	992.7
22	C_58_H_111_NO_21_	+ H_2_O_2_	C22:0-OH	1180.75	14.2	17.4	1018.7
23	C_58_H_111_NO_20_	+ H_2_O	C22:0-OH	1164.76	14.6	17.7	1002.71
24	C_60_H_113_NO_20_	+ H_2_O_2_	C24:1	1190.77	14.6	17.8	1028.72
25	C_58_H_111_NO_19_	+ H_2_O	C22:0	1148.76	15	18	986.71
26	C_60_H_113_NO_19_	+ H_2_O	C24:1	1174.78	15	18	1012.73
27	C_58_H_109_NO_20_	+ O	C22:0-OH	1162.74	15.8	18.6	1000.69
28	C_60_H_111_NO_19_	- H_2_	C24:0-OH	1172.76	16.3	25	1010.71
29	C_58_H_109_NO_19_	N/A	C22:0-OH	1146.75	16.5	25	984.7
30	C_58_H_109_NO_18_	N/A	C22:0	1130.75	16.8	25	968.7
31	C_60_H_111_NO_18_	N/A	C24:1	1156.77	16.9	25	994.72
32	C_60_H_115_NO_21_	+ H_2_O_2_	C24:0-OH	1208.78	17.4	25	1046.73
33	C_60_H_115_NO_20_	+ H_2_O	C24:0-OH	1192.79	17.7	25	1030.74
34	C_60_H_115_NO_19_	+ H_2_O	C24:0	1176.8	17.8	25	1014.75
35	C_60_H_113_NO_20_	+ O	C24:0-OH	1190.77	18	25	1028.72
36	C_60_H_113_NO_19_	N/A	C24:0-OH	1174.78	18	25	1012.73
37	C_60_H_113_NO_18_	N/A	C24:0	1158.78	18.6	25	996.73
IS	C_53_H_99_NO_18_	N/A	C17:0	1060.67	7	13	898.63

### Measurement of lyso-Gb3 and its analogues by means of nano-LC-MS/MS

For sensitive quantification of lyso-Gb3 and its analogues, we used a nano-LC-MS/MS assay system comprising an Ultimate 3000RSLCnano (Thermo Fisher Scientific, Inc., Waltham, MA, USA) and a PAL HTS XT-CTC autosampler (CTC Analytics AG, Zwingen, Switzerland) according to a previous report [16]. The samples were injected via a 1 μL nanoViper Loop (Thermo Fisher Scientific). A Zorbax 300SB-C18 nano column (150 mm x 0.1mm, 3 mm particles; Agilent Technology, Inc., Santa Clara, CA, USA) was used for chromatographic separation of lyso-Gb3 and its analogues. Solvent A comprised 0.2% FA/5% ACN and Solvent B 0.2% FA/ACN. A mobile-phase gradient was produced during a 25 min run: 0 min, 1% B; 10 min, 99% B; 16 min, 99% B; 16.1 min, 1% B; and 25 min, 1% B. The flow rate was 0.5 μL/min. A Q-Exactive mass spectrometer (Thermo Fisher Scientific) was used for the detection of lyso-Gb3 and its analogues. Instrument calibration was performed before each analysis. The targeted MS/MS analysis (HRPS) mode was selected for quantification of lyso-Gb3 and its analogues. The first quadrupole was operated at 1.0 FWHM and the Orbitrap spectrometer at 17,500 FWHM. The AGC target value was set at 1E5, with a maximum injection time of 100 ms. The collision energy value was 25% for all the compounds of interest. The target masses were m/z 786.4482 for lyso-Gb3 and m/z 790.470 for lyso-Gb3-IS. In the case of measurement of lyso-Gb3 analogues, m/z 758.417 for lyso-Gb3(-28), m/z 774.412 for lyso-Gb3(-12), m/z 784.433 for lyso-Gb3(-2), m/z 800.427 for lyso-Gb3(+14), m/z 802.443 for lyso-Gb3(+16), m/z 804.459 for lyso-Gb3(+18), m/z 820.454 for lyso-Gb3(+34), and m/z 836.449 for lyso-Gb3(+50) were used for the targeted MS/MS analysis. The calculation was performed using Quan Browser software (Thermo Fisher Scientific). Higher intensive fragment ions were selected for quantification, i.e., m/z 282.278 for lyso-Gb3, m/z 286.301 for lyso-Gb3-IS, m/z 254.248 for lyso-Gb3(-28), m/z 270.243 for lyso-Gb3(-12), m/z 280.263 for lyso-Gb3(-2), m/z 278.248 for lyso-Gb3(+14), m/z 280.263 for lyso-Gb3(+16), m/z 318.300 for lyso-Gb3(+18), m/z 334.295 for lyso-Gb3(+34), and m/z 350.290 for lyso-Gb3(+50).

### Statistical Analysis

The statistical significance test was performed with Stat Preclinica SAS 9.2 (Takumi Information Technology Inc., Tokyo, Japan). Data are expressed as means ± standard deviation. The differences in the Gb3 and lyso-Gb3 levels in organs and plasma between the *GLA* knockout mice and the wild-type ones were assessed by means of Student’s t test.

## Results

### Identification of MS peaks

The theoretical masses of Gb3 isoforms, having various fatty acids linked with various sphingosine moieties corresponding to lyso-Gb3 and its four major analogues (lyso-Gb3(-2), lyso-Gb3(+16), lyso-Gb3(+18) and lyso-Gb3(+34)) [16], were targeted (Fig 1B). Fragment ions due to neutral loss of a single galactosyl fragment (162.05 Da) from the precursor ion, not fragment ions from a ceramide moiety, were mainly observed with our instrument. Thus, we used them for quantification of Gb3 isoforms. We found that the ceramide moiety of Gb3 isoforms influenced the elution order of different molecular species, i.e., the molecules having short, unsaturated and hydroxylated fatty acids linked with sphingosine were eluted earlier (S1 Fig). From this, we determined the elution time corresponding to the MS peak of each Gb3 isoform. We set the acquisition time window for each compound of interest so as to obtain enough data points. The target masses and acquisition times are shown in Table 1. Some peaks were considered to be mixtures of two or more structural isomers having the same molecular mass.

### Contents of total Gb3 and its isoforms in organs and plasma

As shown in Table 2, the concentration of Gb3 in the kidneys was apparently higher than those in the heart and liver in both the wild-type and *GLA* knockout mice. The mean Gb3 concentrations in all the organs and plasma of the *GLA* knockout mice were significantly higher than those in the wild-type mice (p<0.01).

**Table 2:** Gb3 and lyso-Gb3 concentrations in the wild-type and *GLA* knockout mice.

Samples	Gb3 Wild-Type (N = 3)	Gb3 *GLA* KO (N = 6)	Lyso-Gb3 Wild-Type (N = 3)	Lyso-Gb3 *GLA* KO (N = 6)	Lyso-Gb3/Gb3 (x1,000) Wild-Type (N = 3)	Lyso-Gb3/Gb3 (x1,000) *GLA* KO (N = 6)
Heart (ng/mg tissue) Males	0.86 ± 0.21	970 ± 230	0.00059 ± 0.00026	0.57 ± 0.08	0.75 ± 0.4	0.62 ± 0.20
Heart (ng/mg tissue) Females	1.0 ± 0.3	1300 ± 200	0.00051 ± 0.00016	0.93 ± 0.17	0.60 ± 0.43	0.69 ± 0.11
Kidney (ng/mg tissue) Males	120 ± 20	9800 ± 2800	0.0028 ± 0.0007	1.7 ± 0.1	0.025 ± 0.007	0.20 ± 0.09
Kidney (ng/mg tissue) Females	70 ± 29	14000 ± 4000	0.0018 ± 0.0005	3.3 ± 0.1	0.029 ± 0.009	0.25 ± 0.08
Liver (ng/mg tissue) Males	1.2 ± 0.4	5000 ± 1500	0.011 ± 0.002	6.3 ± 0.5	9.0 ± 1.1	1.3 ± 0.3
Liver (ng/mg tissue) Females	1.8 ± 1.4	6800 ± 1800	0.0084 ± 0.0009	10 ± 1	6.6 ± 3.8	1.6 ± 0.5
Plasma (ng/μL) Males	0.016 ± 0.004	4.4 ± 1.6	0.00067 ± 0.00012	0.19 ± 0.01	42 ± 3	52 ± 21
Plasma (ng/μL) Females	0.018 ± 0.002	2.8 ± 0.5	0.00045 ± 0.00007	0.24 ± 0.01	25 ± 5	81 ± 9

**Fig 2:**
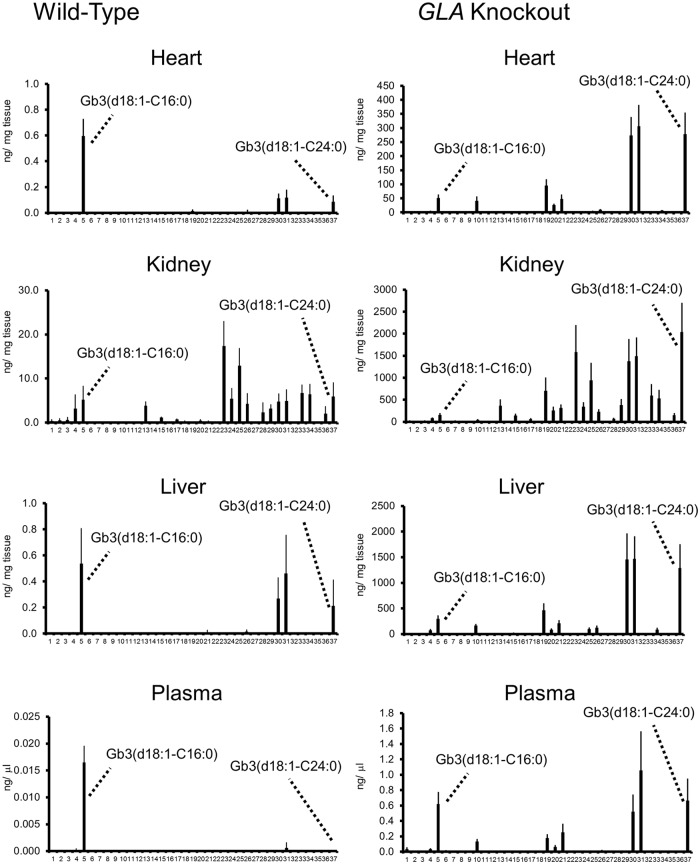
The distributions of Gb3 isoforms in organs and plasma of the wild-type and *GLA* knockout mice. Numbers along the x-axis are elution orders from the RP column and correspond to numbers in Table 1.

The distributions of Gb3 isoforms differs from organ to organ. Various Gb3 isoforms were observed especially in the kidneys (Fig 2), and kidney-specific Gb3 isoforms were hydroxylated. In the *GLA* knockout mice, the concentrations of hydrophobic isoforms (e.g., Gb3(d18:1-C24:0)) in the organs were higher than those of hydrophilic Gb3 isoforms (e.g., Gb3(d18:1-C16:0)). However, in the plasma, the concentrations of hydrophobic Gb3 isoforms were not much higher than those of hydrophilic Gb3 isoforms. On the other hand, in the wild-type mice, the concentrations of hydrophilic isoforms were apparently high in the plasma.

### Contents of lyso-Gb3 and its analogues in organs and plasma

**Fig 3:**
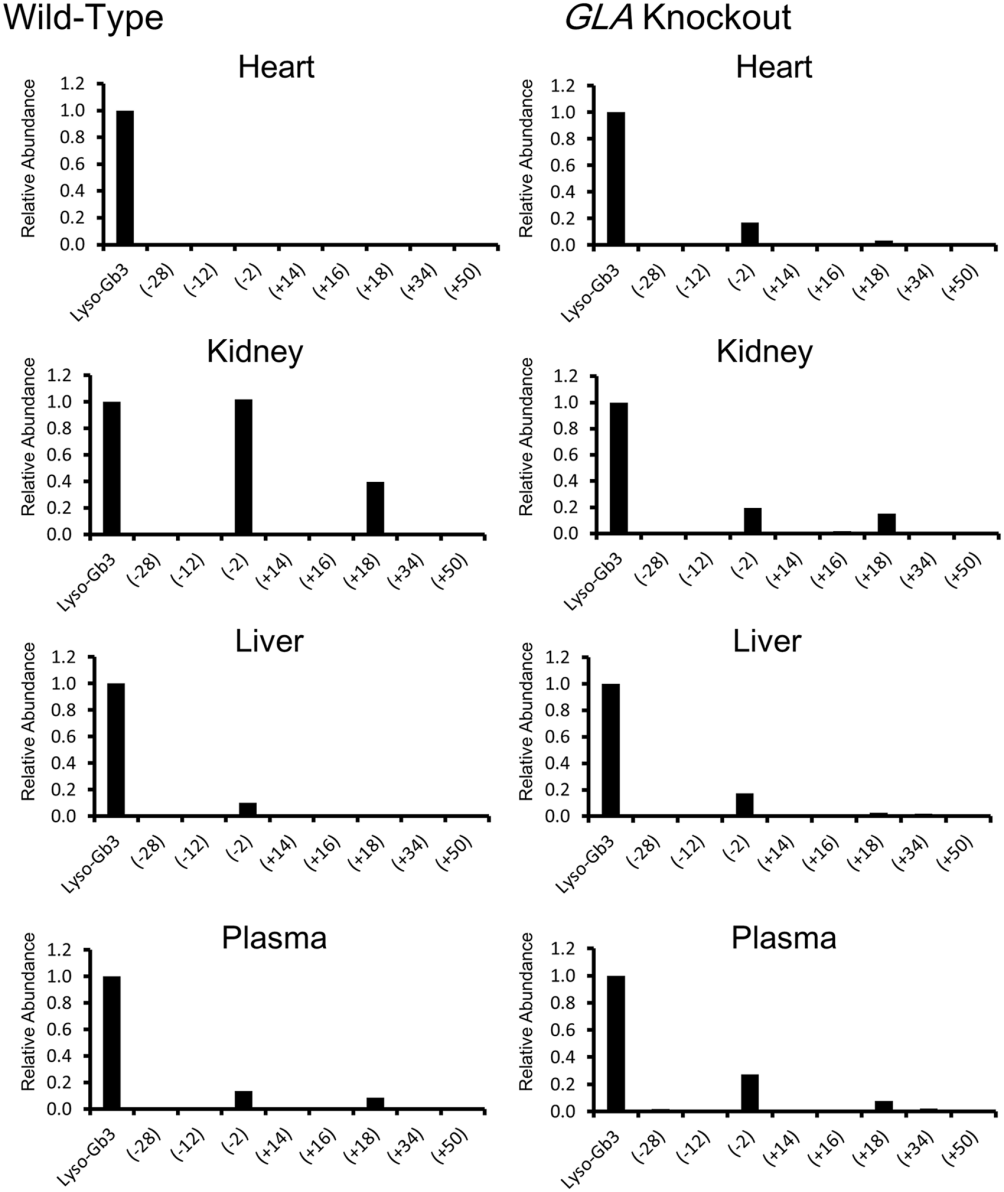
The relative abundance of lyso-Gb3 analogues as to lyso-Gb3 in organs and plasma of the wild-type and *GLA* knockout mice.

The lyso-Gb3 concentration was measured by means of nano-LC-MS/MS. Lyso-Gb3 in all the organs and plasma of the wild-type mice was successfully detected by means of this sensitive method (Table 2). Unlike that of Gb3, the concentration of lyso-Gb3 was high in the liver but not in the kidneys. The mean lyso-Gb3 concentrations in all the organs and plasma of the *GLA* knockout mice were significantly higher than those in the wild-type mice (all, p<0.01). The lyso-Gb3 to Gb3 ratio in the plasma was higher than those in the organs in both the wild-type and *GLA* knockout mice. The concentration of lyso-Gb3 was higher than those of its analogues in all the organs and plasma from both the wild-type and *GLA* knockout mice. Among the lyso-Gb3 analogues, the concentrations of lyso-Gb3(-2) and lyso-Gb3 (+18) were higher than those of the others (Fig 3).

## Discussion

To elucidate the basis of Fabry disease based on accumulated substrates of GLA, we examined the distributions of Gb3 isoforms and lyso-Gb3 and its analogues in the liver, kidneys, heart and plasma of 8-month-old *GLA* knockout mice and compared them with in the case of wild-type ones. It is reported that the Gb3 level of the organs in *GLA* knockout mouse increases up to 20-weeks of age and is stable after 20 weeks of age [26]. Therefore, 8-month-old *GLA* knockout mouse is well-suited for examining the glycosphingolipids distribution in the stationary phase. The results revealed that the contents of these substrates in the organs and plasma from the *GLA* knockout mice were apparently higher than those in the wild-type ones. Especially in the kidneys, which is one of the most affected organs in this disease, the accumulation of a large amount of Gb3 was found.

As to the distributions of Gb3 isoforms, organ specificity was found. In the kidneys, various kinds of hydroxylated residues were found in both the wild-type and *GLA* knockout mice. Such a characteristic distribution of Gb3 isoforms in the kidneys may be due to the organ-specific biosynthetic pathway for Gb3.

In the organs of the *GLA* knockout mice, the proportion of hydrophobic Gb3 isoforms was apparently higher than that in the wild-type mice, suggesting that excessive accumulation of such hydrophobic Gb3 isomers affects the organ and tissues. On the other hand, in plasma, the content of hydrophilic residues was high. The lyso-Gb3 to Gb3 ratio in plasma was also higher than those in organs. It is known that a part of Gb3 synthesized in the liver is transported to the plasma in combination with low-density and high-density lipoproteins [1]. The results of this study suggest that hydrophilic molecules are liable to leak from the liver into the plasma.

The concentration of lyso-Gb3 was apparently higher than those of its analogues in the organs and plasma from both the wild-type and GLA knockout mice. Our previous study revealed that the levels of lyso-Gb3(-2) and lyso-Gb3(+34) were higher than those of the other lyso-Gb3 analogues in plasma from Fabry patients [16]. However, the present study revealed that, in the GLA knockout mice, the concentrations of lyso-Gb3(-2) and lyso-Gb3(+18) were higher than those of the other lyso-Gb3 analogues. This would be due to a difference between humans and mice.

In this study, we elucidated the distributions of Gb3 isoforms and lyso-Gb3 and its analogues in wild-type and *GLA* knockout mice. This information will be useful for elucidating the basis of Fabry disease.

## Supporting Information

S1 Fig MS peaks of Gb3 isoforms. Peak numbers correspond to analytes in Table 1. Some peaks were considered to be mixtures of two or more structural isomers, e.g., Gb3(d18:1-C22:1) and Gb3(d18:2-C22:0).
(TIF)
