# Supplementary figures and images for: Distributions of Globotriaosylceramide Isoforms, and Globotriaosylsphingosine and Its Analogues in an α-Galactosidase A Knockout Mouse, a Model of Fabry Disease
Source: PLoS One. 2015 Dec 14;10(12):e0144958. doi: 10.1371/journal.pone.0144958 (PMC4685999; doi:10.1371/journal.pone.0144958)

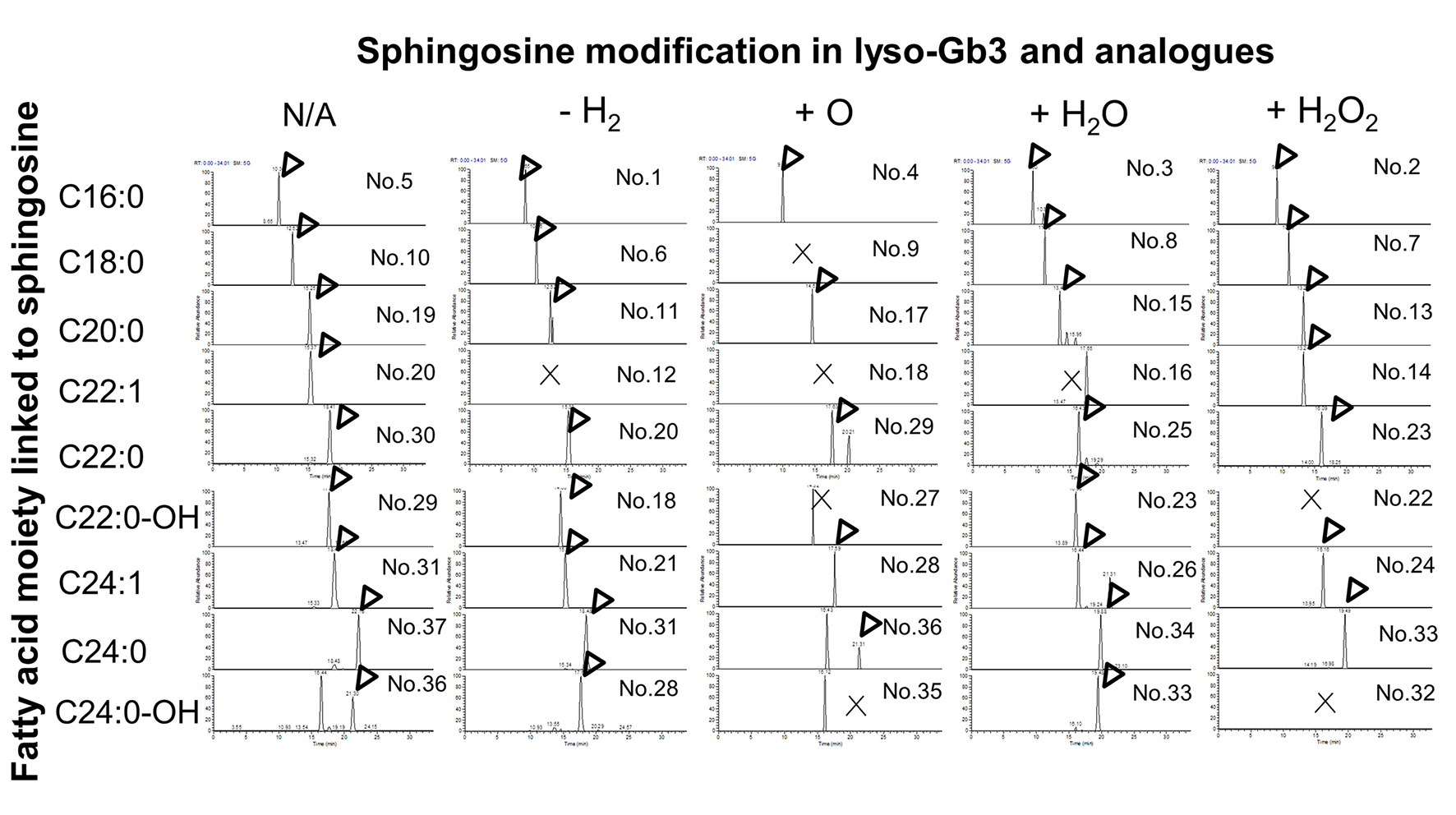

Supplement: S1 Fig — Peak numbers correspond to analytes in Table 1. Some peaks were considered to be mixtures of two or more structural isomers, e.g., Gb3(d18:1-C22:1) and Gb3(d18:2-C22:0). (TIF) [file pone.0144958.s001.tif]
